# Development of reverse transcription loop-mediated isothermal amplification-based assay for rapid and specific detection of human fungal pathogen, *Candida auris*

**DOI:** 10.22034/cmm.2024.345284.1572

**Published:** 2024-11-14

**Authors:** Ankush Kaushik, Zeeshan Fatima, Saif Hameed

**Affiliations:** 1 Amity Institute of Biotechnology, Amity University Haryana, Gurugram (Manesar)-122413, Haryana, India

**Keywords:** *Candida auris*, Loop-mediated isothermal amplification, RT-LAMP, Sensitivity, Specificity

## Abstract

**Background and Purpose:**

Due to the ability of *Candida auris*, a multidrug-resistant human fungal pathogen, to colonize the skin and hospital surfaces, it is pertinent to control its nosocomial outbreaks through rapid diagnosis. Delayed and improper diagnosis of *C. auris* due to misidentification becomes a major hurdle in the prevention of employment of efficient therapeutics leading to the development of drug resistance. The culture-based methods are slow and less sensitive while PCR-based methods are costly. Loop-mediated amplification (LAMP) is a feasible alternative, but it fails to differentiate between live and dead cells. Therefore, this study aimed to evaluate the diagnostic efficiency of the reverse transcription (RT) LAMP approach and compare it with that of the LAMP assay for the detection of *C. auris*.

**Materials and Methods:**

RT-LAMP method was developed for the detection of *C. auris* and its clinical isolates. The limit of detection (LOD), sensitivity, and specificity were evaluated for the developed method using culture RNA. The RT-LAMP reaction for *C. auris* detection was standardized using the primers of a specific 869-bp DNA segment (accession no. XM_018317007), encoding a pyruvate: ferredoxin oxidoreductase domain, from the genome of *C. auris*.

**Results:**

The LOD for the RT-LAMP method was 1ag contrary to 10fg for LAMP method using DNA. Specificity was 100% as determined using a gram-negative bacteria and several other *Candida* species. The RT-LAMP method was intraspecific and displayed no cross reaction even with closely related *Candida* species. The RT-LAMP method was validated on 10 clinical isolates of *C. auris* and showed 100% concordance with a culture-based method.

**Conclusion:**

The RT-LAMP-based method in the present study offered a proof of concept that warrants clinical validation on a large number of samples. Therefore, its diagnostic potential for the rapid, sensitive, and specific detection of *C. auris* could be further exploited in resource-limited regions.

## Introduction

In recent times, fungal pathogenic infections have become an issue of major concern among highly susceptible populations worldwide. One of the major pathogenic fungi of the critical group is *Candida auris* reported in their Fungal Priority Pathogen List provided by the World Health Organization (WHO) published in 2022 [ 1 ]. Discovered in 2009, the first recorded instance of *C. auris* infection occurred in a Japanese woman, whose ear infection was identified through detailed analysis of ribosomal DNA sequencing, specifically targeting the ribosomal DNA D1/D2 and internal transcribed spacer (ITS) regions [ 2 ]. *Candida auris* has high mortality rates and has led to several outbreaks in healthcare facilities around the globe [ 3 ]. It has been reported that most of the *C. auris* isolates are multidrug-resistant, thereby, limiting the therapeutic options [ 4 ]. Furthermore, their ability to persist on surfaces and a lack of accurate methods to prevent misidentification with other *Candida* spp. are the major causes of the outbreaks [ 5 ]. *Candida auris* is commonly misidentified as *C. haemulonii*/ *C. duobushaemulonii*, *C. glabrata*, *C. kefyr*, *C. guilliermondii*, *C. famata*, *C. conglobata*, and *C. utilis* when detected by the Chromagar method [ 6 , 7 ]. Similarly, it is often misidentified as *C. haemulonii*, *C. famata*, and *C. lusitaniae* when detected through the Vitek 2 YST system [ 8 ]. Under such pressing circumstances, rapid but accurate diagnosis could be the key to efficient management of the available therapeutic options and prevention of drug resistance onset.

Various methods have been developed to distinguish *C. auris* from other closely related *Candida* species, including Chromagar, mass spectrometry, polymerase chain reaction (PCR) sequencing, and recombinase polymerase amplification [ 6 , 7, 9, 10 ]. However, each method has its limitations, such as misinterpretation with closely related species, requirement for specialized equipment, or being time-consuming and expensive. Current diagnoses available in the market are PCR-based or mass-spectrometry-based, which are very expensive and not available in tier 2 and tier 3 cities. Culture-based methods are cheap; however, they are not specific as they are unable to differentiate between other *Candida* species. 

Loop-mediated isothermal amplification (LAMP) is a nucleic acid sequence amplification technique introduced in 2000 [ 11 ]. LAMP is a popular new technology for rapid nucleic acid detection and is efficiently used for the detection of human pathogens (viruses, fungi, bacteria, and malaria) [ 12 - 15 ]. Additionally, LAMP fails to differentiate between live and dead cells. Contrary, reverse transcription LAMP (RT-LAMP) utilizes RNA as a template where the reverse transcriptase activity of Bst polymerase converts RNA into cDNA and the reaction follows the same way as in LAMP [ 16 ]. RNA is transient and short-lived in nature inside the cell; hence, only live and actively metabolizing cells will have a high content of RNA (i.e., in various forms alternatively splicing variants, multiple transcribed copies from genomic DNA). The RT-LAMP not only helps to determine the content of viable cells within a culture, reducing the possibility of false positives but also increases the sensitivity of the assay for detection [ 17 ]. Therefore, the present study aimed to add to the existing literature about the utilization of the RT-LAMP method for the detection of *C. auris* which is more sensitive than LAMP using DNA as a template.

## Materials and Methods

### Materials

All media chemicals yeast extract, peptone and dextrose were obtained from Himedia (Mumbai, India). Nucleic acid isolation kit was obtained from Qiagen, Germany. Moreover, the Warmstart master mix was obtained from NEB, England. Nuclease-free water was obtained from Thermo Fischer Scientific, USA, and the primers were synthesized from Eurofilms.

### Candida growth conditions

The *Candida* strains were cultured in yeast extract peptone dextrose (YPD) broth with the composition of yeast extract 1% (w/v), dextrose 2% (w/v), and peptone 2% (w/v). All *Candida* strains were stored in 30% (v/v) glycerol stocks at -80 °C. The cells were revived freshly at 30°C on YPD broth before each experiment to ensure the restoration of the strains. *Candida auris* CBS10913T (Clade II) [ 18 ] strain was used in all the experiments and if any other strains were used it is mentioned in the figure legend.

### Nucleic acid isolation

*Candida auris* was inoculated in YPD at 30 °C overnight in 50 ml media. Culture of the log phase was used for DNA and RNA isolation. The DNA was isolated by using a DNA extraction kit from Qiagen as described in the manufacturer protocol. Similarly, RNA was isolated using the RNA extraction kit from Qiagen as described in the manufacturer protocol. After isolation of DNA and RNA, both were treated with RNase or DNase respectively to remove any other contamination, checked on 2% gel electrophoresis for integrity, and estimated for concentration via nanodrop.

### RT- LAMP primer design

To formulate the LAMP primers, within the genome of *C. auris*, a specific 869-bp DNA segment (accession no. XM_018317007), encoding a pyruvate: ferredoxin oxidoreductase (PFOR) domain, was identified with limited similarity to other *Candida* species. Furthermore, using Primer Explorer V5 software (accessible at https://primerexplorer.jp/lampv5e/index.html) and NEB LAMP primer design tool, a set of LAMP primers was designed (Table 1) targeting a 192-bp fragment (encompassing bp 774 to 965 of the XM_018317007 sequence) [ 13 ]. 

**Table 1:** List of primer sequences used for the study

Primer	Sequence (5ʹ→3ʹ)
FIP	AGGCTACTGAGCTTGCTGGTGTAACCAAACCAACAGGAGAGG
BIP	ACGGTTTCAGGGTTAGCATGGCTCAACAAAGTCGCTGGTACA
Loop-F	CATCTCGAAGGCCTCGGT
Loop-B	CACATACTCGAACGGAGTC
F3	GGGAAAGGAACCCTGACCT
B3	GGACACAGCATTCGAAGTGT

### LAMP and RT-LAMP reaction

A total volume of 12.5 μl was used to set the LAMP and RT-LAMP reactions. The reaction was prepared by using 6.25 μl of 2X WarmStart Colorimetric Master mixes, 1.25 μl of 10X Primer mix containing RT-LAMP primers, and 1 μl of purified DNA or RNA with 2.5 μl of nuclease-free water. The reaction was set for 40 min at 65 °C. This RT-LAMP system is designed to provide fast and clear visual detection of amplification based on the production of protons and the subsequent drop in pH that occurs from the extensive DNA polymerase activity in an RT-LAMP reaction, producing a change in solution color from pink to yellow [ 12 ].

## Results

### Standardization of RT-LAMP reaction for C. auris detection

**Figure 1:**
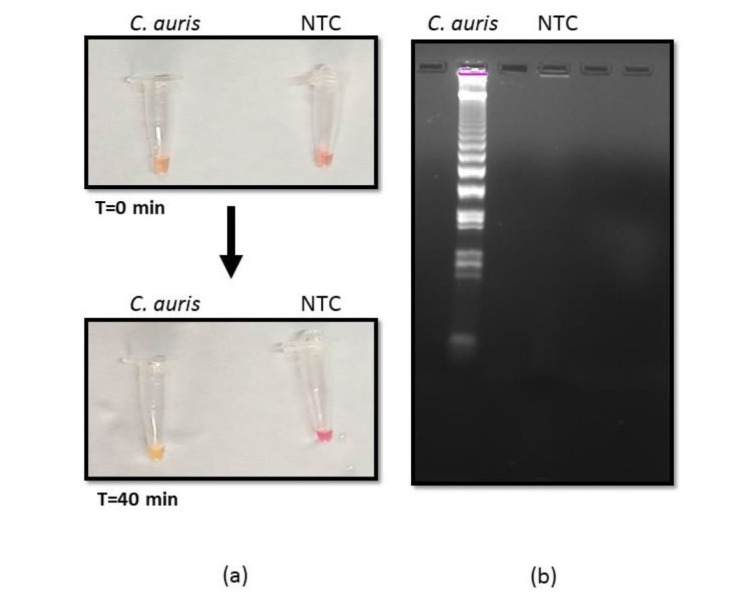
Optimization reverse transcription loop-mediated amplification (RT-LAMP) (a) RT-LAMP reaction with *Candida auris* RNA and negative template control visualized by color change from pink to yellow after 40 min at 65 ℃. (b) Agarose gel image of the amplified product obtained from LAMP reaction.

First, we sought to evaluate the feasibility of using RT-LAMP to detect the presence of *C. auris*. This was achieved by targeting the PFOR gene with the primers as described in the methods section (Table 1). It was observed by the naked eye that amplification was achieved owing to a color change from pink to yellow in the presence of *C. auris* RNA, contrary to no color change in negative template control (NTC) (Figure 1a). The positive amplification reaction was also validated by running the amplicons on 2% gel electrophoreses where typical smear banding pattern confirmed successful amplification (Figure 1b).

### Analytical sensitivity of the RT-LAMP reaction

**Figure 2:**
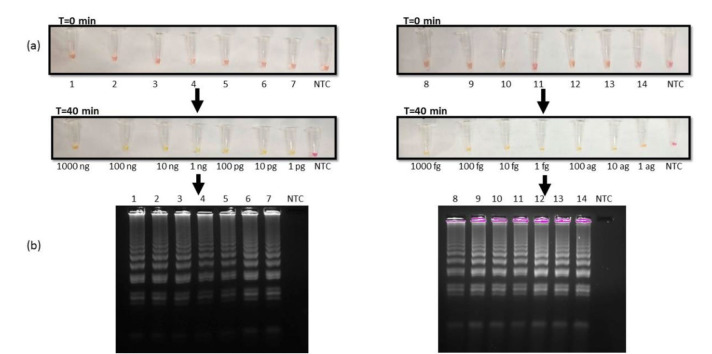
Limit of detection of reverse transcription loop-mediated amplification (RT-LAMP) reaction (a) RT-LAMP reaction in tubes 1-14 with RNA concentrations: Lane 1)1000 ng, 2)100 ng, 3)10 ng, 4)1ng, 5)100 pg, 6)10 pg, 7)1 pg (left panel) 8)1000 fg, 9)100 fg, 10)10 fg, 11)1 fg, 12)100 ag, 13)10 ag, 14)1 ag (right panel), visualised by color change from pink to yellow after 40 min at 65 ℃. (b) Agarose gel images of amplified products obtained from RT-LAMP reactions.

Different dilutions of extracted RNA from pure cultures of *C. auris* were used to determine the limit of detection (LOD) for our assay. The LOD was defined as the lowest dilution of RNA that was detectable by our assay with the naked eye. Firstly, the serial dilutions were tested with RNA concentrations ranging from 1,000 ng to 1 pg. It was found that the color change was noticeable at 1pg RNA concentration (Figure 2a; left panel). Furthermore, the researchers diluted the RNA until 1 ag concentration and again observed that color change was detectable even at 1 ag concentration (Figure 2a; right panel). Therefore, the LOD of the developed assay that was clearly visible both with the naked eye and gel electrophoresis (Figure 2b) was 1 ag of RNA.

**Figure 3:**
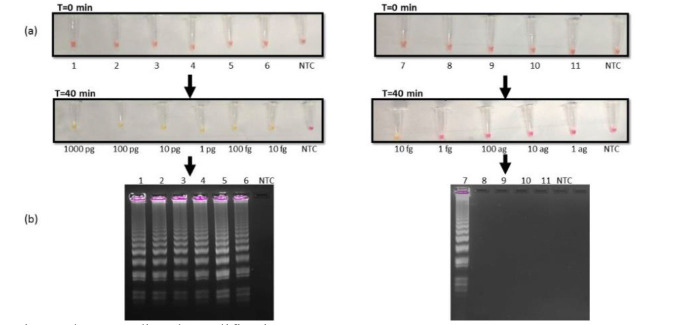
Lane 1)1000 pg, 2)100 pg, 3)10 pg, 4)1pg, 5)100 fg, 6)10 fg, (left panel), 7)10 fg, 8)1 fg, 9)100 ag, 10)10 ag, 11) 1 ag (right panel), visualized by colour change from pink to yellow after 40 minutes at 65℃. (b) Agarose gel images of amplified products obtained from LAMP reactions.

Additionally, this study sought to compare the analytical sensitivity of our newly developed RT-LAMP assay with the LAMP reaction using DNA as a template. This was achieved using different dilutions of extracted DNA from pure cultures of *C. auris*. Firstly, the serial dilutions were tested with DNA concentrations ranging from 1,000 pg to 10 fg. It was found that the reaction was sensitive at 10fg DNA concentration (Figure 3a; left panel). Furthermore, the researchers diluted the DNA until 1 ag concentration and observed that color change was still detectable only at 10 fg concentration (Figure 3a; right panel). These observations were also validated on gel electrophoresis (Figure 3b). Therefore, the LOD of the LAMP assay that was visible both with the naked eye and gel electrophoresis was 10 fg of DNA.

### Specificity of RT- LAMP assay for C. auris detection

**Figure 4:**
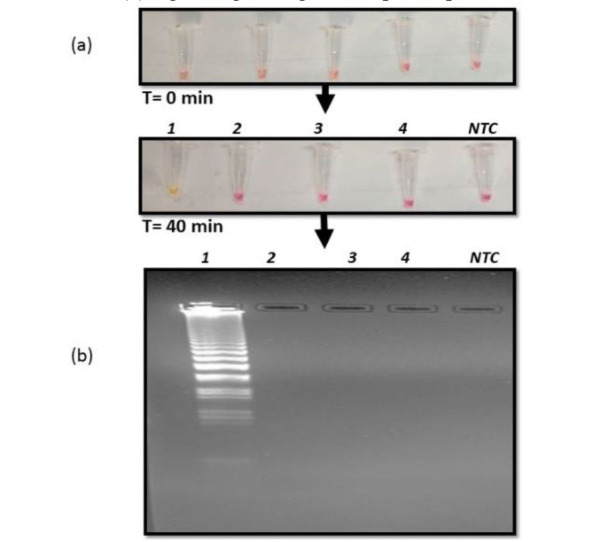
Interspecies specificity of reverse transcription loop-mediated amplification (RT-LAMP) reaction (a) RT-LAMP reaction of culture RNA extracted from Lane 1) *C. auris*, 2) *C. albicans*, 3) *Mycobacterium smegmatis* and 4) *E coli*, visualized by color change from pink to yellow after 40 minutes at 65℃. (b) Agarose gel image of the amplified product obtained from RT-LAMP reaction.

**Figure 5:**
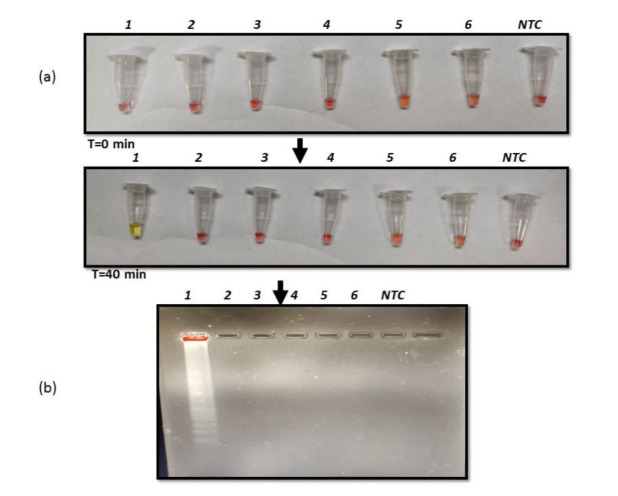
Intraspecies specificity reverse transcription loop-mediated amplification (RT-LAMP) reaction (a) RT-LAMP reaction of culture RNA extracted from lane 1) *Candida auris*, 2) *Candida albicans*, 3) *Candida glabrata*, 4) *Candida tropicalis*, 5) *Candida parapsilosis*, and 6) *Candida krusei.*, visualized by color change from pink to yellow after 40 min at 65 °C. (b) Agarose gel image of the amplified product obtained from RT-LAMP reaction.

To evaluate the specificity of the assay in this study, different strains were used, viz., *Mycobacterium smegmatis*, *C. albicans*, and *Escherichia coli* other than *C. auris*. The RT-LAMP reaction was performed at 65 ºC for 40 min using template RNA isolated from *Mycobacterium marinum*, *M. smegmatis*, *C. auris*, and *E. coli* cultures. It was found that there was a clear amplification of only *C. auris* target PFOR gene as detected by naked eye owing to color change from pink to yellow contrary to retention of pink color in *M. smegmatis*, *C. albicans*, *E. coli*, and NTC (Figure 4a). This result was validated by gel electrophoresis of the amplicons depicting typical smear pattern only for *C. auris* (Figure 4b). Furthermore, intra-species specificity was also evaluated for the developed assay. Related *Candida* strains of *C. auris*, *C. albicans*, *C. glabrata*, *C. tropicalis*, *C. parapsilosis*, and *C. krusei* were included in the reaction. To our expectations, it was found that the amplification was achieved only with *C. auris* RNA which displayed yellow color contrary to all other *Candida* spp. RNA which retained their pink color (Figure 5a). Gel electrophoresis of the amplicons further validated the above-mentioned observations (Figure 5b).

### Clinical validation of RT- LAMP assay for C. auris detection

**Figure 6:**
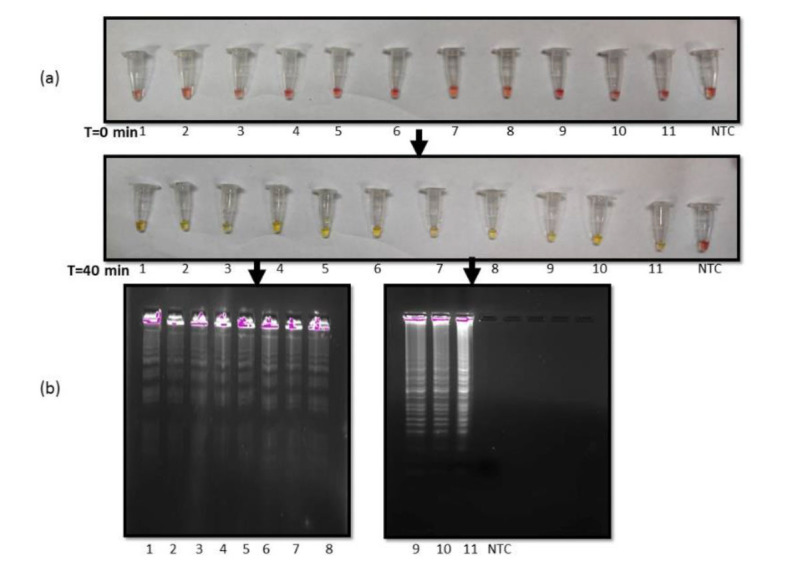
Clinical validation of reverse transcription loop-mediated amplification (RT-LAMP) for *Candida auris* (a) RT-LAMP reaction of culture RNA extracted from clinical isolates of *C. auris* in lanes 1) 1622, 2) 218834, 3) 1251, 4) C22/6089, 5) 37229, 6) 202916, 7) 202982, 8) P1188, 9) C-33, 10) C-34, 11) CBS10913T, and 12) NTC. (b) Agarose gel image of the amplified product obtained from RT-LAMP reaction.

Validation of the developed assay was performed at the pilot level on 10 clinical isolates of *C. auris*. A clear amplification was found in all the tested *C. auris* strains by the naked eye owing to color change from pink to yellow contrary to the retention of pink color in NTC (Figure 6a). This result was again validated by gel electrophoresis of the amplicons depicting typical smear pattern for all tested isolates of *C. auris* (Figure 6b).

## Discussion

The rise in fungal infections among susceptible populations has brought *C. auris* as a rising obstacle against efficient therapeutics. In 2022, WHO declared *C. auris* a critical pathogen due to the complexities in its diagnosis leading to misidentification and treatment delays [ 1 ]. In contrast to laboratory testing, point-of-care testing offers a faster turnaround resulting in better clinical and economic outcomes as it overcomes the time-consuming steps of sample collection, transport, and processing by bringing the laboratory close to the patient. Rapid and accurate diagnosis with LAMP has emerged as a promising cost-effective method to detect *Candida* species due to its capability to detect visually, rapidly, and specifically [ 13 , 19 , 20 ]. The LAMP-based methods are much more sensitive and specific in comparison to culture and matrix-assisted laser desorption ionization-time of flight-based methods available in clinics for the diagnosis of *C. albicans* [ 21 ]. LAMP method was also able to detect vulvovaginal candidiasis with high sensitivity in a recent study [ 22 ]. Another study utilized the LAMP method to detect *Candida* with a sensitivity of 100% by using the blood sample from a patient [ 20 ]. The LAMP method was also used to detect *C. glabrata* with much sensitivity as compared to the culture method [ 23 ]. The novel LAMP diagnosis method was used for specific diagnosis of fungal species, such as *Aspergillus fumigatus* [ 24 , 25 ]. 

In recent years, a few variants of LAMP have also emerged which include RT-LAMP and multiplex LAMP (M-LAMP) [ 26 ]. The RT-LAMP method has been utilized for the detection of SARS-CoV-2 and is considered equivalent to RT-PCR in terms of utility [ 12 , 27 ]. Similarly, RT-LAMP has been used for the detection of parasites, such as amoeba and malaria [ 28 , 29 ]. In fact, the versatility of RT-LAMP has recently been reviewed for the early detection of pathogens [ 30 ]. Hence, the current study aimed to utilize the RT-LAMP approach to demonstrate its higher sensitivity and specificity, thereby, allowing faster detection and discrimination of *C. auris* from other microorganisms.

The present study used the PFOR domain of the *C. auris* genome that shared limited similarity with other *Candida* species based on the literature [ 12 ]. The specific primers were designed (Table 1) as described in the methods section and the RT-LAMP reaction was optimized at the temperature of 65 ºC for 40 min using RNA as template. The color change was detected by the naked eye and the typical gel electrophoresis pattern confirmed the successful amplification (Figure 1). Next, it was sought to determine the LOD by estimating the analytical sensitivity of the RT-LAMP assay. It was found that our assay was sensitive enough to give positive results till 1ag of RNA (Figure 2). Furthermore, the RT-LAMP was compared with the DNA LAMP method, and it was observed that unlike RNA, when DNA was used as a template, the LOD remained 10 fg (Figure 3). This confirmed that RT-LAMP was more sensitive than LAMP. This could also be attributed to the fact that RNA is abundant in copy numbers, including the splicing variants, compared to DNA; hence, it enables detection from metabolically active live cells contrary to dead cells. Another reason could be the single-chain RNA structure, which is more prone to degradation but less susceptible to contamination than DNA [ 17 , 31 ]. The Enhanced sensitivity of RT-LAMP can hold promise for rapid diagnosis of *C. auris* infections which are prone to misdiagnosis. 

Furthermore, the specificity of the developed method was assessed using *M. smegmatis*, *E. coli*, and *C. albicans* strains. To our expectation, the RT-LAMP method could specifically discriminate *C. auris* from other tested strains (Figure 4). Additionally, this method was also deployed to test the intraspecies specificity and it was observed that color change could be detected only with *C. auris* RNA, contrary to other *Candida* strains (Figure 5). Based on these results, it was further sought to use the clinical isolates of *C. auris* to validate the assay. For this reason, 10 clinical isolates of *C. auris* were utilized. As it was expected, the researchers observed the color changes from pink to yellow for all tested clinical isolates of *C. auris*, contrary to NTC which remained pink (Figure 6a) as also apparent from gel electrophoresis (Figure 6b). This is also evident from the fact that LAMP-based methods are 10-100 times more sensitive, compared to the conventional PCR assays and other isothermal amplification techniques, such as CPA, PSR, or HAD [ 32 ]. The developed method in this study offers few advantages. For instance, the results can be interpreted with the naked eye owing to color change which makes the method suitable for limited-resource settings. Additionally, it is not dependent on equipment as it can be performed at a single temperature; therefore, these methods are cost-effective.

## Conclusion

To win the battle against the disease, especially in resource-limited regions, simple, cost-effective, and rapid diagnostic methods are required for clinical application. In the era of isothermal-based amplification methods for disease diagnosis, the present study, although still in its infancy, offered considerable proof of concept for RT-LAMP-based methods that could be helpful in the rapid identification of *C. auris* infections. Development and improvement of these methods to meet the diagnostic gap in developing countries requires further investigation.
